# Optimization of Sensory Quality and Short-Term Colloidal Stability of *Akebia trifoliata* Cloudy Juice for Beverage Development

**DOI:** 10.3390/foods15162911

**Published:** 2026-08-20

**Authors:** Haoqing Wen, Yue Li, Md. Nasir Hossain Sani, Jean Wan Hong Yong, Junyang Song

**Affiliations:** 1 College of Landscape Architecture and Art, Northwest A&F University (NWAFU), Yangling 712100, China; wenhaoqing2000@163.com (H.W.); yueli2399@outlook.com (Y.L.); 2 Department of Biosystems and Technology, Swedish University of Agricultural Sciences (SLU), 234 56 Alnarp, Sweden; jean.yong@slu.se; 3 Brightlands Future Farming Institute, Faculty of Science and Engineering, Maastricht University, 5911 Venlo, The Netherlands

**Keywords:** *Akebia trifoliata*, cloudy juice, novel fruit, sensory evaluation, beverage processing

## Abstract

*Akebia trifoliata* is an underutilized fruit with distinctive sensory characteristics and potential for beverage development; however, its application is limited by flavor-balance and suspension-stability challenges. This study optimized the formulation and short-term physical stability of *A. trifoliata* cloudy juice using single-factor tests, orthogonal experiments, and fuzzy mathematical sensory evaluation. Each treatment was independently prepared in triplicate (*n* = 3), with each preparation considered an experimental replicate. The effects of pulp, sugar, and citric acid concentrations on sensory quality were evaluated, with citric acid identified as the dominant factor affecting overall acceptability. The highest-performing basic formulation among the tested combinations contained 14% pulp, 8% sugar, and 0.05% citric acid, yielding a cloudy juice with characteristic aroma, smooth texture, and balanced sweet–sour taste. To improve short-term suspension stability, pectin, guar gum, xanthan gum, sodium alginate, and sodium carboxymethyl cellulose were assessed. Pectin showed the strongest individual stabilizing effect, although excessive viscosity reduced sensory acceptability. Orthogonal optimization identified a compound stabilizer system of 0.03% pectin, 0.05% guar gum, and 0.06% xanthan gum as the formulation providing the most favorable balance between the suspension stability coefficient and trained-panel sensory score among the tested combinations. The fuzzy mathematical evaluation approach provided a structured framework for integrating multiple sensory and stability-related indicators into formulation decisions. These findings provide a practical basis for the formulation and short-term stability screening of *A. trifoliata* cloudy juice as a value-added beverage and support broader utilization of this indigenous fruit resource. Long-term storage stability, microbial safety, bioactive properties, rheological behavior, and consumer acceptance require further evaluation.

## 1. Introduction

The increasing demand for natural, nutritious, and health-oriented beverages has stimulated scientific and industrial interest in underutilized fruit resources. Such fruits provide opportunities to diversify raw materials, expand value-added food products, and introduce new sensory characteristics and product-development opportunities [1,2]. Among these resources, *Akebia trifoliata*, commonly known as three-leaf akebia or chocolate vine, is a perennial liana of the Lardizabalaceae family that is widely distributed in East Asia, particularly in Japan and the Qinba Mountains region of China [3]. Although the plant has a long history of use in traditional medicine, its edible fruit has only recently attracted attention in food science because of its distinctive composition, flavor, and potential for product development [4].

The pulp of *A. trifoliata* fruit contains sugars, organic acids, flavonoids, triterpenoid saponins, polyphenols, and other bioactive constituents that may contribute to its nutritional and functional value [5]. In addition, its volatile aroma compounds and polysaccharide-rich matrix provide a characteristic flavor profile and mouthfeel, indicating its suitability as a raw material for juice and beverage processing [6,7]. Despite these advantages, *A. trifoliata* remains largely underexploited in commercial beverage applications. This limited utilization is mainly attributable to insufficient information on processing suitability, flavor adjustment, colloidal stability, and sensory standardization [7]. Therefore, systematic formulation and stabilization studies are needed to support the development of A. trifoliata-based beverages and improve the value-added utilization of this indigenous fruit resource.

Cloudy juice is a promising product form for *A. trifoliata* because it retains insoluble pulp particles, colloidal polysaccharides, and phenolic components that are often reduced during juice clarification [8]. In cloudy juice production, fruits or vegetables are processed without complete removal of suspended solids, allowing the beverage to retain a fuller mouthfeel, natural appearance, and a greater proportion of pulp-associated constituents [9]. Compared with clarified juices, cloudy juices are often associated with enhanced freshness perception and improved retention of phytochemicals [10]. However, the same suspended particles and complex colloidal structures that contribute to these desirable properties can also cause sedimentation, phase separation, enzymatic browning, and aroma deterioration during processing and storage [11,12]. These quality defects reduce consumer acceptability and limit industrial application. For *A. trifoliata* juice, the stabilization challenge may be particularly important because its phenolic compounds, pulp particles, and polysaccharide systems can interact dynamically and affect both sensory quality and physical stability [8].

Hydrocolloid stabilizers are commonly used to improve the stability of cloudy fruit beverages by increasing viscosity, enhancing particle suspension, and reducing sedimentation. Stabilizers such as sodium carboxymethyl cellulose, xanthan gum, pectin, guar gum, and sodium alginate have been applied in fruit-based beverage systems to improve physical stability, texture, and mouthfeel [13,14,15,16]. However, stabilizer performance depends strongly on type, concentration, and interaction with the juice matrix. Excessive addition may increase viscosity beyond an acceptable level, mask characteristic fruit flavor, or create an undesirable mouthfeel. Therefore, stabilizer selection should not be based only on physical stability but should also consider sensory acceptability. Optimization approaches, including single-factor experiments, orthogonal design, response surface methodology, and other multivariate methods, have been widely used to balance formulation variables and improve the quality of fruit-based products [17,18]. Nevertheless, no systematic formulation or compound-stabilizer optimization strategy has been reported for *A. trifoliata* cloudy juice.

Sensory evaluation is particularly important in the development of beverages from novel or unfamiliar fruits because consumer acceptance depends not only on nutritional value but also on aroma, taste, color, texture, and overall palatability. Conventional sensory scoring methods provide useful information, but they are often affected by evaluator subjectivity, individual preference, and uncertainty in describing complex sensory attributes [19,20,21]. This limitation is more pronounced for uncommon fruits such as *A. trifoliata*, for which panelists may lack a familiar reference standard to evaluate its characteristic aroma, sweet–sour balance, and mouthfeel [22]. Fuzzy mathematical sensory evaluation offers a structured method for addressing this problem by transforming qualitative sensory judgments into quantitative membership values [21,23,24]. By integrating multiple weighted indicators, fuzzy evaluation can provide a more systematic basis for formulation decisions [25,26]. This approach has therefore been increasingly applied in food sensory evaluation and product optimization [27,28].

Accordingly, the present study aimed to optimize the sensory quality and short-term suspension stability of *A. trifoliata* cloudy juice using single-factor experiments, orthogonal design, and fuzzy mathematical sensory evaluation. The effects of pulp, sugar, and citric acid concentrations on sensory acceptability were first investigated to determine the highest-performing basic formulation among the tested combinations. Subsequently, individual and compound stabilizers were evaluated to improve colloidal stability while maintaining desirable sensory properties. This study provides a practical formulation and short-term stability-screening strategy for the development of a stable, sensory-acceptable *A. trifoliata* cloudy juice and contributes to the broader utilization of underexploited fruit resources in value-added beverage applications.

## 2. Materials and Methods

### 2.1. Fruit Material and Cloudy Juice Preparation

**Figure 1:**
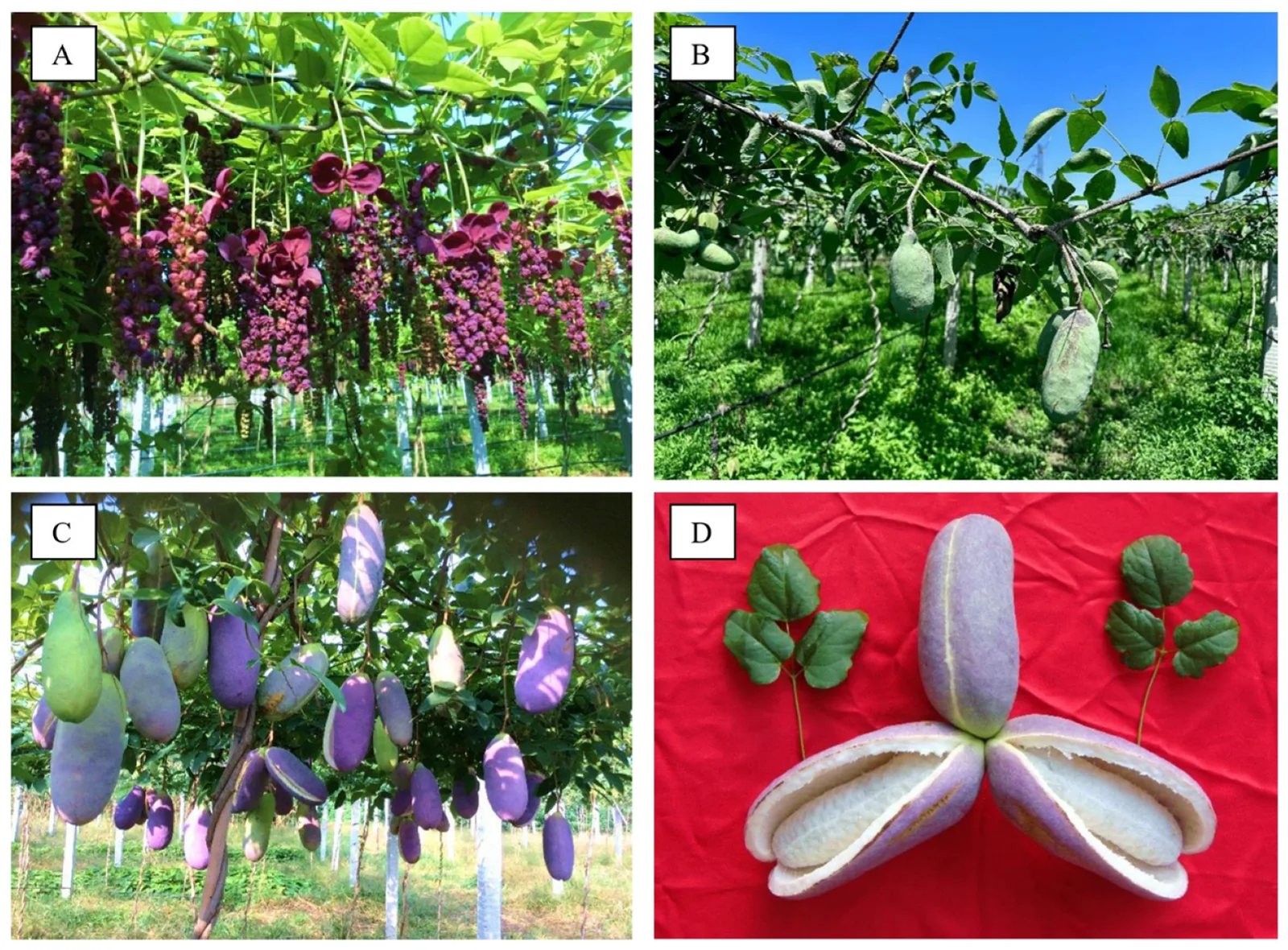
Morphological and cultivation characteristics of *Akebia trifoliata* var. “Qinbao 1” at the August Honey Wild Fruit and Tree Research Institute, Huyi District, Xi’an, Shaanxi Province, China. (**A**) Leaves and flowers; (**B**) developing fruits and climbing growth habit supported by trellis structures; (**C**) ripening fruits; (**D**) close-up view of ripening fruits and seeds.

*Akebia trifoliata* (Thunb.) Koidz. var. “Qinbao 1” (Lardizabalaceae) fruits were obtained from the August Honey Wild Fruit and Tree Research Institute, Huyi District, Xi’an, Shaanxi Province, China (108°37′ E, 34°07′ N; 418.8 m above sea level) (Figure 1). The plants were cultivated under conventional orchard management practices, with trellis structures used to support their climbing growth habit. Fruits were harvested in September 2023 from five-year-old plants at the commercial ripening stage, which was determined based on external peel coloration, pericarp dehiscence, and pulp softness. After harvest, fruits were immediately transported to the laboratory of Northwest A&F University under cooled conditions and stored at −80 °C until further processing.

Before juice preparation, fruits were thawed under controlled conditions, manually sorted to remove damaged samples, washed thoroughly, and peeled. The pulp was separated from seeds using a custom-built pulp separator and homogenized to obtain raw pulp. The pulp was then diluted with distilled water at a ratio of 1:1 (*v*/*v*) and filtered through cloth meshes of different pore sizes. The most suitable mesh size was selected based on short-term suspension stability. The basic formulation consisted of *A. trifoliata* pulp, sucrose, citric acid, and distilled water. Preliminary flavor optimization was performed using single-factor experiments followed by an orthogonal experimental design, and sensory quality was evaluated using a fuzzy mathematical sensory evaluation method. Stabilizers, including pectin, guar gum, xanthan gum, sodium alginate, and sodium carboxymethyl cellulose (CMC-Na), were subsequently screened and optimized to improve short-term suspension stability and trained-panel sensory performance. Ingredient and stabilizer concentrations are expressed as percentages of the final juice formulation (*m*/*v*).

For preparation of the final cloudy juice, the optimized formulation was filtered through a 400-mesh cloth, as selected in the mesh-size screening experiment, and filled into pre-sterilized glass bottles. The bottles were loosely capped, degassed in boiling water for 5–6 min, hermetically sealed while hot, and subjected to secondary sterilization at 100 °C for 15 min. After sterilization, the bottles were shaken gently to ensure uniform dispersion and cooled to room temperature to obtain the prepared cloudy juice formulations.

### 2.2. Optimization of the Basic Cloudy Juice Formulation

The basic formulation of *A. trifoliata* cloudy juice was optimized using a two-step procedure consisting of single-factor experiments and an orthogonal experimental design. In the single-factor experiments, the effects of pulp content, sucrose concentration, and citric acid concentration on sensory quality were evaluated under controlled formulation conditions. Pulp content was first tested at 10%, 12%, 14%, 16%, and 18%, while sucrose and citric acid concentrations were fixed at 7% and 0.10%, respectively. Sucrose concentration was then evaluated at 5%, 6%, 7%, 8%, and 9%, with pulp content and citric acid concentration fixed at 12% and 0.10%, respectively. Finally, the citric acid concentration was tested at 0.05%, 0.10%, 0.15%, 0.20%, and 0.25%, while pulp content and sucrose concentration were maintained at 12% and 7%, respectively. Each treatment was independently prepared in triplicate (*n* = 3).

Sensory quality was assessed using the fuzzy mathematical comprehensive evaluation method, which converted panel ratings for multiple sensory attributes into a weighted composite score [23,24,26]. Based on the single-factor results, an L9(3^4^) orthogonal experimental design was used to determine the highest-performing tested combination of pulp, sucrose, and citric acid. The selected factor levels were pulp content at 12%, 14%, and 16%; sucrose concentration at 6%, 7%, and 8%; and citric acid concentration at 0.05%, 0.10%, and 0.15%. The orthogonal design allowed for efficient evaluation of the relative effects of formulation variables while reducing the number of experimental runs required for optimization [29]. Sensory scores obtained from the fuzzy mathematical evaluation were used as the response values for comparison of the tested formulations.

### 2.3. Short-Term Suspension Stability and Stabilizer Optimization

The stability of *A. trifoliata* cloudy juice was evaluated through a stepwise procedure involving mesh-size screening, individual stabilizer selection, single-factor stabilizer optimization, and a compound-stabilizer orthogonal design. Raw pulp was diluted with distilled water at a ratio of 1:1 (*v*/*v*) and gently stirred at approximately 200 rpm for 5 min. The diluted pulp was filtered once through woven filter cloths of 100, 200, 300 and 400 mesh to obtain cloudy juice samples with different particle-size distributions. According to the U.S. Standard Sieve Series, these mesh sizes correspond to nominal aperture diameters of approximately 150, 75, 50, and 37 µm, respectively [30]. The filtrates were prepared using constant pulp, sucrose, and citric acid concentrations, then evaluated for suspension stability to identify the most suitable filtration mesh for beverage preparation [31].

Individual stabilizer screening was performed by adding pectin, guar gum, xanthan gum, sodium alginate, or CMC-Na to the base cloudy juice at 0.10% (m/v). After static storage at 22 ± 1 °C for three days, the samples were compared based on suspension stability and sensory acceptability. The three stabilizers with superior performance—pectin, guar gum, and xanthan gum—were selected for further optimization. These stabilizers were considered suitable because of their thickening capacity, suspension-stabilizing capabilities, and potentially complementary functions in acidic fruit beverage systems. The three-day assessment was used only for short-term formulation screening and was not intended to represent product shelf life.

Single-factor stabilizer trials were then conducted within practical solubility and viscosity ranges. Guar gum and xanthan gum were each tested at 0.05%, 0.10%, 0.15%, 0.20%, 0.25%, and 0.30% (m/v), while pectin was tested at 0.025%, 0.050%, 0.075%, 0.100%, 0.125%, and 0.150% (m/v). The prepared formulations were stored at 22 ± 1 °C for three days, after which suspension stability and sensory quality were evaluated. The optimal concentrations identified from the single-factor tests were used as the basis for the construction of a compound-stabilizer L9(3^4^) orthogonal design. Factor levels were set at 20%, 30%, and 40% of the corresponding single-factor optimum concentrations. Accordingly, the pectin levels were 0.02%, 0.03%, and 0.04%; guar gum levels were 0.05%, 0.075%, and 0.10%; and xanthan gum levels were 0.04%, 0.06%, and 0.08%. The optimal stabilizer combination was determined using suspension stability indices combined with sensory evaluation scores. Each of the nine compound-stabilizer formulations was independently prepared in triplicate (*n* = 3). The highest-performing tested combination was identified using a composite score integrating the suspension stability coefficient and trained-panel sensory score.

### 2.4. Sensory Evaluation and Fuzzy Mathematical Analysis

#### 2.4.1. Panel and Evaluation of Juice Formulations

The sensory quality of *A. trifoliata* cloudy juice formulations was evaluated by a trained panel of 20 members using the fuzzy mathematical composite rating method [22]. The same trained panel was used throughout the formulation and stability evaluations. The sensory attributes for formulation evaluation were color, aroma, taste, and appearance, defined as the factor set expressed as U_1_ = {color, aroma, taste, appearance}. The rating set was V_1_ = {excellent, good, medium, poor}, corresponding to scores of 100, 80, 60, and 40, respectively. Single-factor evaluation matrices were constructed, and a weighted average-type comprehensive judgment model was applied. The weighting set was W_1_ = {0.2, 0.2, 0.4, 0.2}, corresponding to color, aroma, taste, and appearance, respectively. Taste was assigned the highest weighting because it was considered the primary determinant of overall acceptability in fruit juice beverages. The weighting coefficients were defined before analysis, were not estimated from the treatment results, and were applied identically to every formulation.

For each sensory attribute, the proportion of panelists assigning a sample to each of the four rating categories was used as the membership value for that category. These membership values formed the fuzzy evaluation matrix. The attribute-specific membership values were then combined using the predefined weighting coefficients, and the resulting membership vector was converted to a composite sensory score using the 100, 80, 60, and 40 score vectors. This procedure was applied identically to all formulations and permits reproduction of the composite score from the panel-rating frequencies.

All evaluations were conducted in a sensory laboratory at 22 ± 1 °C and 60 ± 5% relative humidity under uniform white lighting. Samples were served at 22 ± 1 °C in identical containers labelled with randomized three-digit codes. The presentation order was randomized independently for each evaluation session, and panelists were not informed of the formulation identities. Because color, homogeneity, sedimentation, and phase separation were explicit evaluation attributes, visual differences were not masked; coding and randomized presentation were instead used to reduce sample-identification, expectation, and order biases. All panelists participated voluntarily and provided informed consent before evaluation. The sensory evaluation criteria for formulation optimization are shown in Table 1. The sensory protocol and institutional permission are described in the Institutional Review Board Statement and supporting Supplementary Material.

#### 2.4.2. Sensory Evaluation of Stability

Stability-related sensory attributes were evaluated using the same 20-member trained panel and fuzzy mathematical composite rating approach. The factor set for stability evaluation was defined as U_2_ = {appearance, mouthfeel, gum-related flavor}, and the rating set was V_2_ = {excellent, good, medium, poor}, corresponding to scores of 100, 80, 60, and 40, respectively. The weighting set was W_2_ = {0.4, 0.3, 0.3}, corresponding to appearance, mouthfeel, and gum-related flavor, respectively. Appearance was assigned the highest weighting because sedimentation, phase separation, and visual homogeneity are critical indicators of cloudy juice stability. The weighting coefficients were defined before analysis and applied consistently to all formulations. Mouthfeel and gum-related flavor were assigned equal weights to account for undesirable thickness and hydrocolloid-related flavor.

The evaluation criteria are presented in Table 1. Evaluations were performed in duplicate, and mean values were used for subsequent analysis. The coefficient of variation among panelists remained below 10%, indicating acceptable consistency in sensory scoring.

### 2.5. Determination of Suspension Stability

Suspension stability was measured using a GENESYS 10S UV-Vis spectrophotometer (Thermo Fisher Scientific, Waltham, MA, USA). Samples were first scanned across the full wavelength range to identify the maximum absorbance, which occurred at 300 nm. Absorbance at 300 nm was selected because turbidity in cloudy beverage systems is highly responsive in the ultraviolet region and reflects light scattering by suspended particles [29]. The absorbance maximum of *A. trifoliata* cloudy juice at this wavelength indicated that particle dispersion contributed substantially to light attenuation.

For stability determination, the absorbance of a well-mixed aliquot of cloudy juice at 300 nm was first recorded before centrifugation as A_0_. A second aliquot from the same independently prepared batch was then centrifuged at 4000 rpm for 10 min, and the absorbance of the supernatant at 300 nm was recorded as A. The stability coefficient (R) was defined as R = A/A_0_, where values closer to 1.0 indicate higher suspension stability and a smaller loss of turbidity following centrifugation. This centrifugation-based coefficient was used as an empirical index for comparison of short-term suspension stability among formulations and was not described as a non-destructive measure or as evidence of shelf life.

### 2.6. Composite Stability Score

To integrate physicochemical and sensory indices, a composite stability score was calculated by combining the suspension stability coefficient and sensory evaluation score, each assigned a weighting of 50%. Indicator affiliation was calculated as

$$\mu =\frac{X-X_{\min}}{X_{\max}-X_{\min}}$$

where μ represents the indicator affiliation, X is the observed value, and X_min_ and X_max_ are the minimum and maximum values of the respective indicator. The two normalized indicator values were then combined at equal weight. This integrated metric was used only to rank the tested formulations according to the balance between short-term suspension stability and trained-panel sensory performance; higher scores indicate more favorable combined performance.

### 2.7. Statistical Analysis

All statistical analyses were performed using R (version 4.3.0). Each treatment consisted of three independently prepared juice batches (*n* = 3), and each independently prepared batch was considered the experimental unit. Duplicate sensory evaluations and any repeated analytical measurements from the same batch were averaged before inferential analysis. Differences among treatments in single-factor experiments, including pulp content, sucrose concentration, citric acid concentration, mesh size, stabilizer type, and stabilizer concentration, were analyzed by one-way analysis of variance (ANOVA). Mean separation was performed using Tukey’s honestly significant difference (HSD) post hoc test. The L9(3^4^) orthogonal designs were analyzed using fixed-effects ANOVA to evaluate the significance and relative contribution of each factor. Statistical significance was defined at *p* < 0.05. Level means (k) and range values (R) were additionally used for descriptive comparison of factor levels. Unless otherwise specified, error bars in figures represent SD calculated from the three independent preparations.

## 3. Results

### 3.1. The Formulation of Akebia trifoliata Cloudy Juice

#### 3.1.1. One-Way Test of Formulation

**Figure 2:**
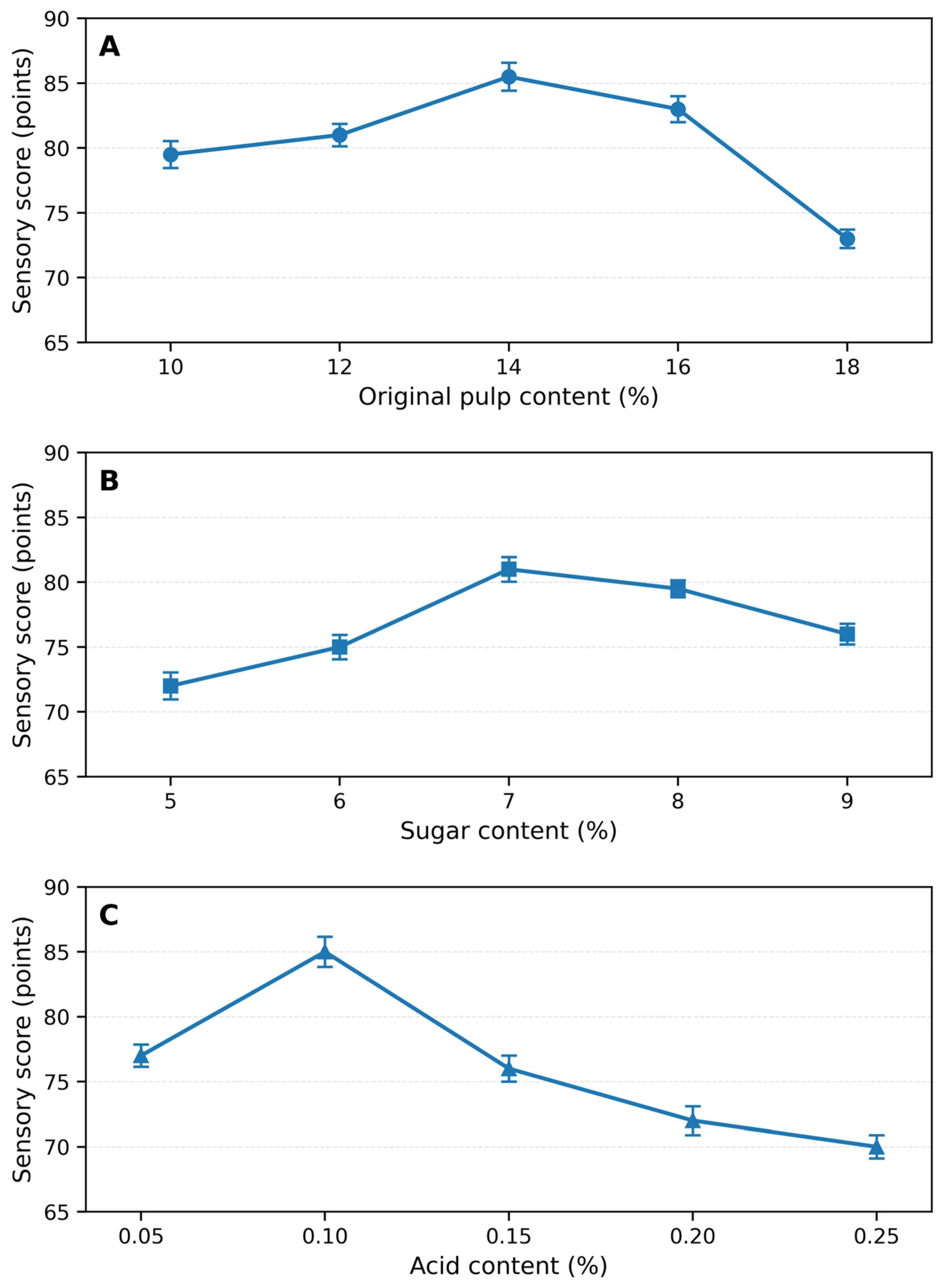
Effects of original pulp (**A**), sugar (**B**) and acid (**C**) contents on sensory evaluation of cloudy juices. Values represent mean ± SE of composite sensory scores from three independent preparations (*n* = 3).

Flavor formulation represents a pivotal determinant of sensory quality in cloudy juice systems, with the relative proportions of pulp, sugar, and acid exerting pronounced effects on the composite sensory score (Figure 2A–C). As illustrated in Figure 2A, variation in pulp concentration markedly influenced the overall composite sensory score. Scores increased steadily from 10% to 14% pulp, reaching a maximum value of 85.2 at 14%, representing the highest composite sensory score within the tested range. Lower composite sensory scores were observed at 10–12% pulp, while concentrations of 16–18% also produced lower scores than 14%. Accordingly, the 12–16% pulp range was selected for subsequent orthogonal testing. The effect of sugar concentration followed a similar unimodal trend (Figure 2B). Sensory scores increased from 5% to 7%, reflecting a progressive enhancement in the equilibrium between sweetness and acidity, and peaked at 7%. At concentrations above 7%, the composite sensory score declined. Thus, sugar concentrations in the range of 6–8% were selected for subsequent orthogonal testing.

The acid concentration also exerted a significant influence on the composite sensory score (Figure 2C). Scores rose with increasing citric acid up to 0.10% and subsequently declined at higher concentrations. At higher concentrations (>0.15%), the composite sensory score decreased further. Consequently, citric acid levels between 0.05% and 0.15% were selected for subsequent orthogonal testing. Collectively, the single-factor experiments delineated effective formulation ranges of 12–16% pulp, 6–8% sugar, and 0.05–0.15% citric acid, which were subsequently applied to orthogonal optimization.

#### 3.1.2. Orthogonal Test of Formulation

The flavor of fruit juices is governed not by a single constituent but by the integrated ratio of its principal components—most notably, the sugar–acid balance. To refine the formulation of *A. trifoliata* cloudy juice, three factors—pulp concentration, sugar concentration, and citric acid concentration—were selected for an orthogonal L9 (3^4^) test based on the results of the single-factor experiments. The factor levels and the composite sensory scores, calculated using the fuzzy mathematics evaluation method, are summarized in Table 2 and Supplementary Table S1.

Analysis of the R-values revealed that the relative influence of the three factors on sensory quality followed the order of citric acid concentration (C) > pulp concentration (A) > sugar concentration (B). Acid level exerted the most pronounced effect on the composite sensory score, underscoring its critical role in modulating the distinctive sweet–sour profile of A. trifoliata. In contrast, sugar concentration had the least impact within the tested range. The k-value analysis provided further insight into the highest-scoring levels of each factor, with rankings of A_2_ > A_1_ > A_3_ for pulp concentration, B_3_ > B_2_ > B_1_ for sugar concentration, and C_1_ > C_3_ > C_2_ for citric acid concentration. These results indicated that 14% pulp, 8% sugar, and 0.05% citric acid yielded the highest average sensory scores among the tested combinations.

The results of ANOVA (Supplementary Table S2) confirmed that citric acid concentration significantly affected sensory ratings (*p* < 0.05), while pulp and sugar concentrations showed no statistically significant effects. Thus, acidity emerged as the principal determinant of the composite sensory response within the tested formulation range. Based on the integration of R-value, k-value, and ANOVA analyses, 14% pulp, 8% sugar, and 0.05% citric acid (A_2_B_3_C_1_) were selected as the highest-performing formulation among the tested combinations. This formulation was subsequently used for the stabilizer-screening experiments.

### 3.2. Short-Term Suspension Stability of Akebia trifoliata Cloudy Juice

#### 3.2.1. Effect of Filtration Mesh on Suspension Stability

**Figure 3:**
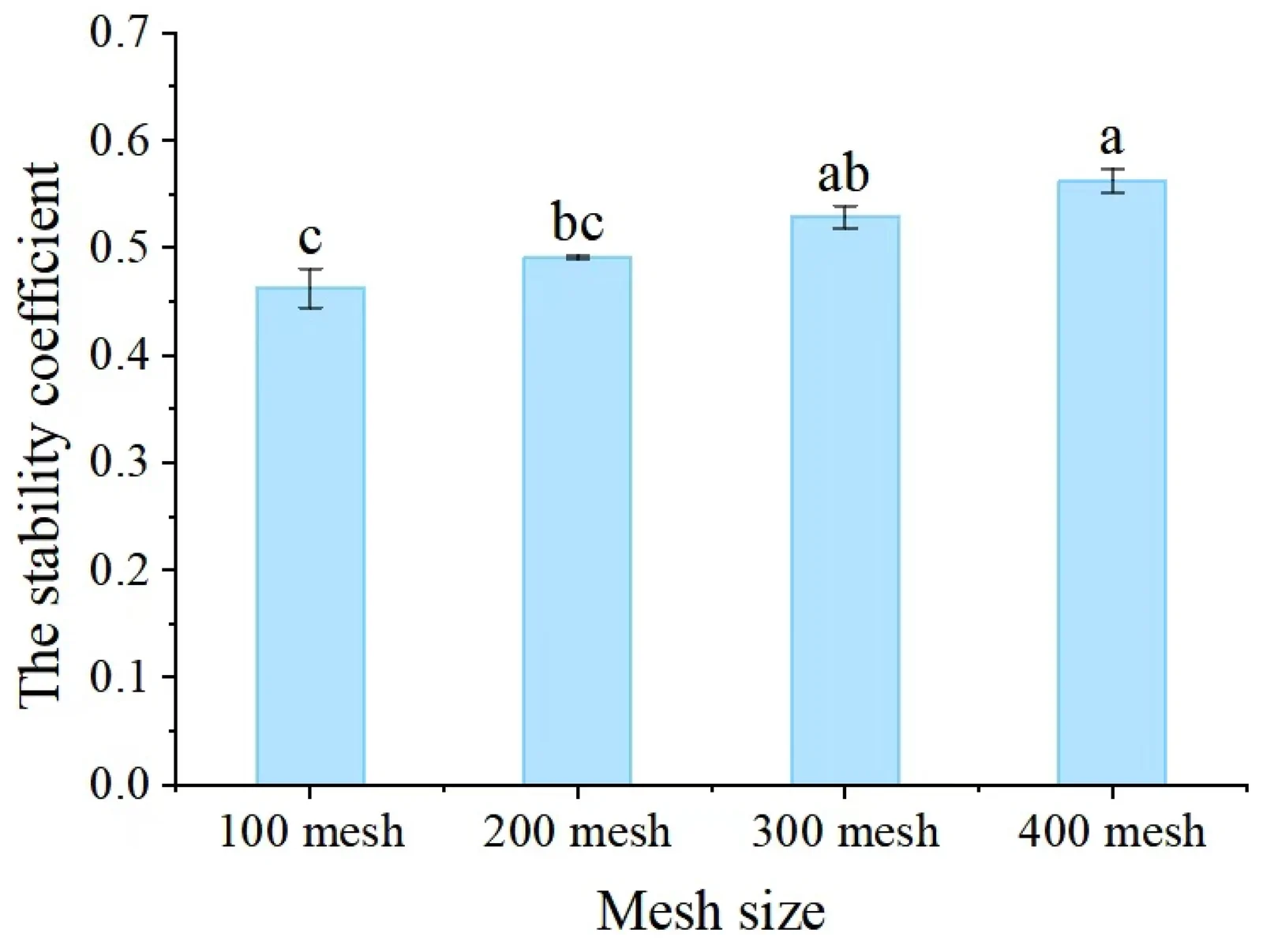
Effects of mesh size on the stability of juices. Values are means ± SE of three independent preparations (*n* = 3). Different letters indicate significant differences among treatments; values not sharing a common letter differ significantly at *p* < 0.05.

The particle size of pulp residues is a critical determinant of cloud stability in fruit-based beverages, as finer particles remain suspended longer and reduce sedimentation [31]. To examine this effect in *A. trifoliata* cloudy juice, the pulp was filtered through mesh sizes ranging from 100 to 400, and suspension stability was subsequently evaluated (Figure 3). The results demonstrate a progressive increase in the stability coefficient with finer filtration, with the highest stability observed at 400 mesh. This pattern is consistent with improved short-term suspension stability under finer filtration. Accordingly, a 400-mesh filter was selected for subsequent juice preparation.

#### 3.2.2. Screening of Stabilizers

**Figure 4:**
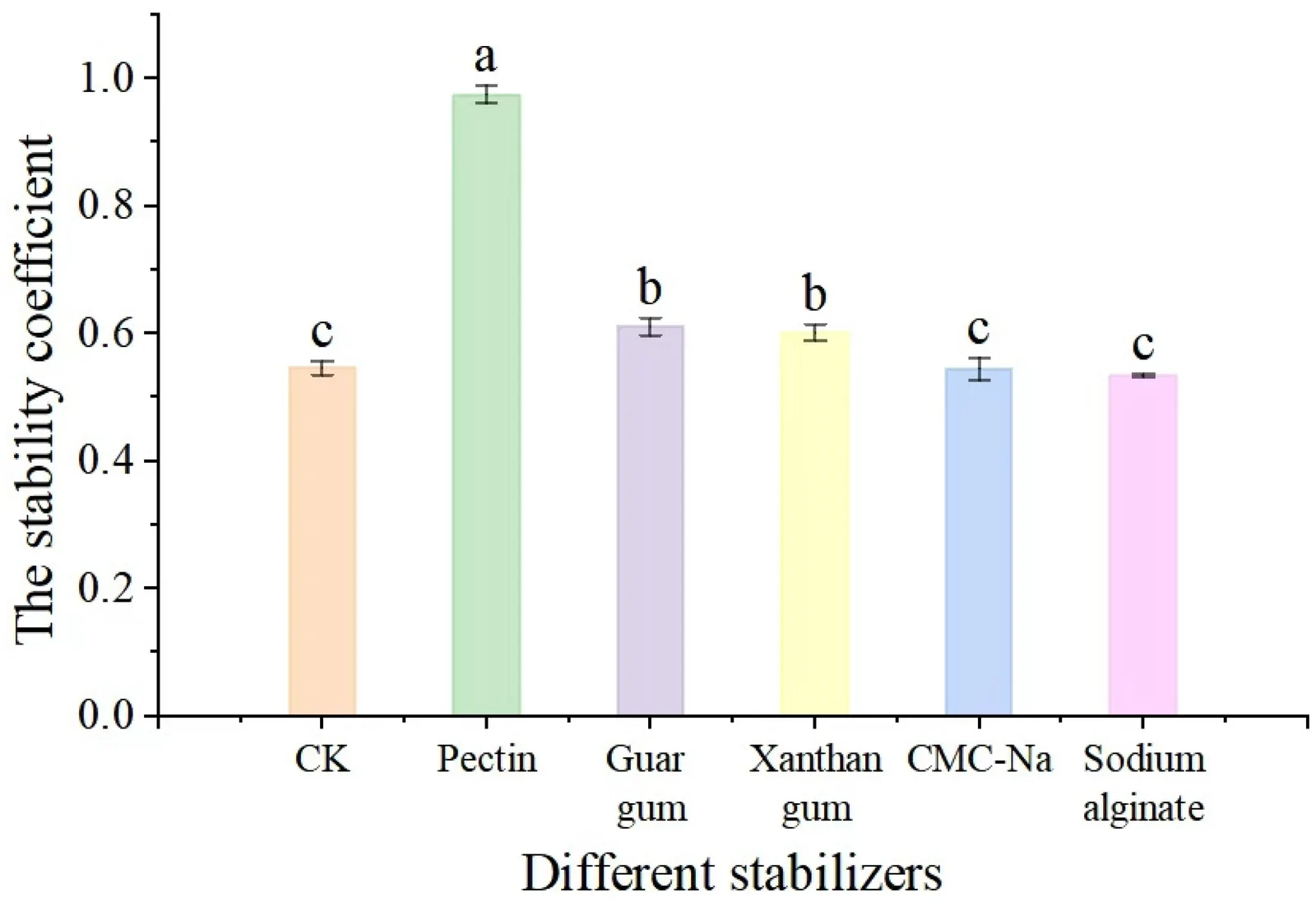
Effects of different stabilizers on the stability of juices. Values are means ± SE of three independent preparations (*n* = 3). Different letters indicate significant differences among treatments; values not sharing a common letter differ significantly at *p* < 0.05.

The addition of stabilizers markedly improved the suspension stability of *A. trifoliata* cloudy juice compared with the control (Figure 4). However, their effectiveness differed considerably. Pectin produced the highest stability coefficient, followed by guar gum and xanthan gum, whereas CMC-Na and sodium alginate showed weaker effects. The overall stabilizing capacity ranked as follows: pectin > guar gum > xanthan gum > CMC-Na > sodium alginate. This ranking refers specifically to the individual-stabilizer screening. Based on their higher suspension stability coefficients, pectin, guar gum, and xanthan gum were selected for further optimization.

#### 3.2.3. One-Way Effects of Stabilizer Concentration

**Figure 5:**
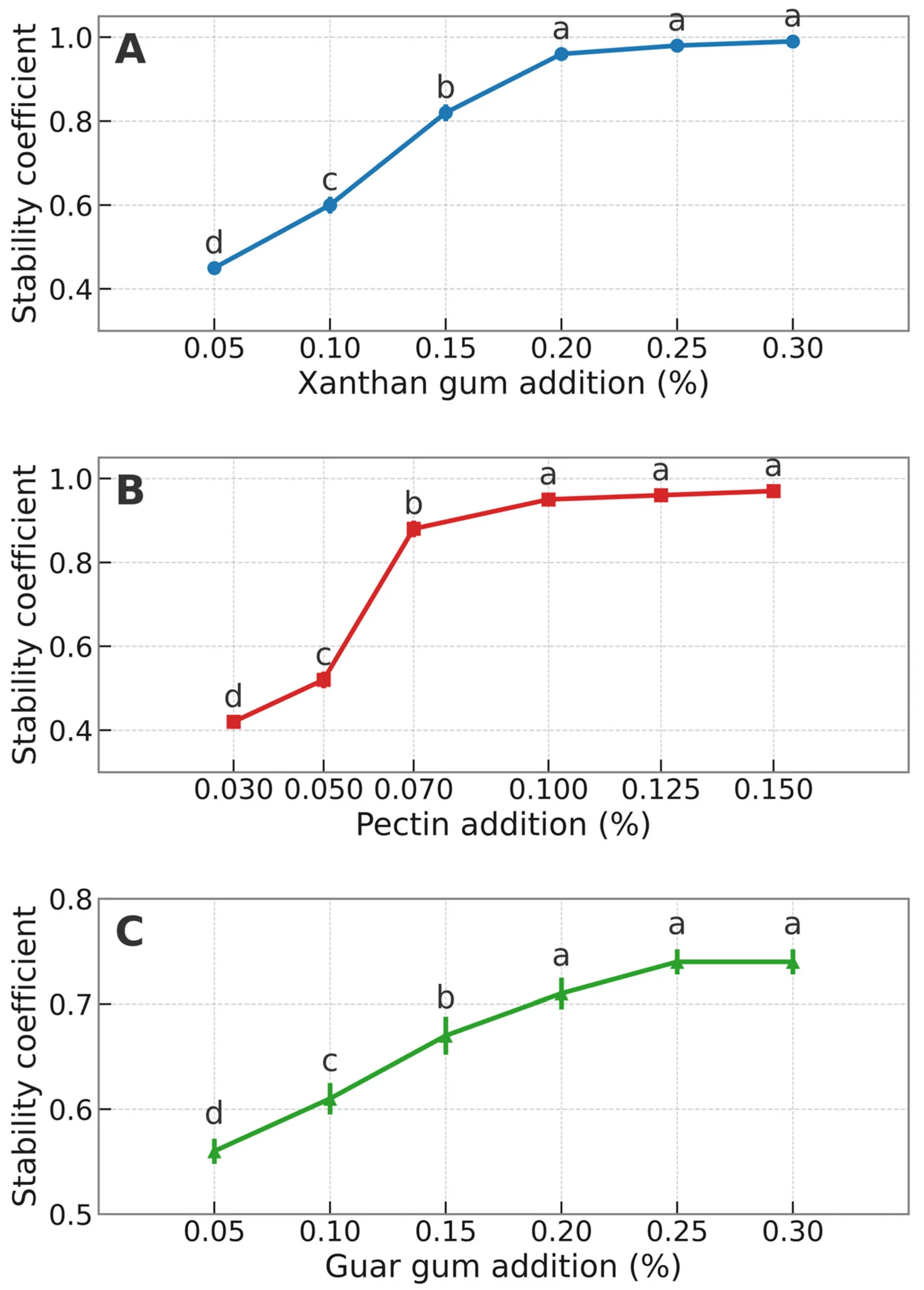
Effects of stabilizer concentration on the suspension stability coefficient of *Akebia trifoliata* cloudy juice: (**A**) xanthan gum; (**B**) pectin; (**C**) guar gum. Values are means ± SD of three independent preparations (*n* = 3). Different letters indicate significant differences among concentrations within each stabilizer according to Tukey’s HSD test (*p* < 0.05).

The concentration of stabilizers had a marked influence on the suspension stability of *A. trifoliata* cloudy juice, with distinct trends observed for xanthan gum, pectin, and guar gum (Figure 5; Supplementary Figure S1). For xanthan gum, stability improved progressively with increasing concentration, with a pronounced increase up to 0.20%, after which only small additional increases were observed. Thus, 0.20% xanthan gum was selected as the single-factor concentration for the construction of the compound-stabilizer design.

For pectin, stability also increased with concentration and plateaued at 0.10%. At this level, the suspension stability coefficient reached 0.975. However, when used alone at 0.10%, the texture of the juice became excessively viscous, detracting from sensory quality. This trade-off supported evaluation of pectin at lower concentrations within a compound-stabilizer system. This limitation highlights the need for compound stabilizer systems, as single-agent pectin is unsuitable despite its strong stabilizing effect. For guar gum, delamination decreased steadily with concentration, and stability reached a plateau at 0.25%. Concentrations above this level provided no additional benefit, and 0.25% guar gum was therefore selected for subsequent compound-stabilizer design. Collectively, the one-way concentration tests identified selected single-factor concentrations of 0.20% xanthan gum, 0.10% pectin, and 0.25% guar gum, which were used to define the factor levels for the subsequent orthogonal experiment.

#### 3.2.4. Orthogonal Optimization of Stabilizers

An orthogonal design was employed to optimize compound stabilizers using pectin, guar gum, and xanthan gum at three graded levels (20%, 30%, and 40% of their respective optimal concentrations; Table 3, Supplementary Table S3). Stability evaluation combined suspension stability coefficients with sensory scores, ensuring that physical stability and trained-panel sensory performance were considered. Because the response variable was the composite stability score, the relative influence of the three stabilizers was interpreted according to the composite-score range analysis presented in Table 3.

Based on the range analysis of the composite stability score, the relative influence of the stabilizers followed the order of xanthan gum (C) > pectin (A) > guar gum (B). This ranking refers to the compound-stabilizer composite response and is therefore distinct from the individual-stabilizer ranking reported in Section 3.2.2. The k-value analysis further revealed the highest composite-score level for each factor, with a ranking of A_2_ > A_1_ > A_3_ for pectin, B_1_ > B_2_ > B_3_ for guar gum, and C_2_ > C_3_ > C_1_ for xanthan gum. The level means identified A_2_B_1_C_2_, corresponding to 0.03% pectin, 0.05% guar gum, and 0.06% xanthan gum, as the selected compound formulation. The error-column range (R = 0.392) exceeded the ranges for the three stabilizer factors, indicating substantial residual variation and warranting cautious interpretation of factor rankings.

Analysis of variance (Supplementary Table S4) indicated that pectin, guar gum, and xanthan gum did not exert statistically significant individual effects on the stabilization of *A. trifoliata* cloudy juice. Among the nine tested combinations, A_2_B_1_C_2_ (0.03% pectin, 0.05% guar gum, and 0.06% xanthan gum) produced the highest composite score (0.951), with a suspension stability coefficient of 0.925 and a trained-panel sensory score of 89.5. Given the non-significant individual factor effects and the relatively large error-column range, A_2_B_1_C_2_ is interpreted as the highest-performing combination within the tested design rather than as a statistically confirmed global optimum.

## 4. Discussion

The development of *Akebia trifoliata* cloudy juice highlights both the potential and the technical challenges of transforming an underutilized fruit into a value-added beverage product. Cloudy fruit juices are complex dispersed systems in which sensory acceptability and physical stability are closely connected. A formulation with desirable flavor may still be rejected if sedimentation, phase separation, excessive viscosity, or poor visual uniformity occurs during storage. Conversely, a highly stable system may not be acceptable if stabilizers create gumminess, mask fruit aroma, or alter the natural mouthfeel. Therefore, successful development of *A. trifoliata* cloudy juice requires simultaneous optimization of flavor balance, pulp content, particle dispersion, and hydrocolloid composition. The present results indicate that these factors collectively influenced trained-panel sensory performance and short-term suspension stability, supporting an integrated formulation strategy rather than optimization of a single quality attribute [32,33].

The results of formulation optimization showed that the citric acid concentration had the greatest influence on sensory quality, followed by pulp concentration and sucrose concentration. This indicates that acidity was the primary driver of the sensory balance of *A. trifoliata* cloudy juice within the tested formulation range. In fruit beverages, acidity contributes not only to sourness but also to freshness perception, aroma release, sweetness modulation, and overall flavor clarity. A moderate level of citric acid can enhance the perception of fruitiness and improve sweet–sour harmony, whereas excessive acidification may generate a sharp taste and suppress the characteristic fruit aroma. Among the tested combinations, the formulation containing 14% pulp, 8% sucrose, and 0.05% citric acid produced the highest composite trained-panel sensory score. This result is consistent with previous reports showing that sugar–acid balance is a key determinant of flavor perception and overall sensory acceptance in fruit juices [32,34].

Pulp concentration also played an important role in determining juice quality. Increasing pulp content can enhance cloudiness, body, aroma intensity, and the perception of naturalness, which are desirable properties in cloudy juice products. However, excessive pulp can increase viscosity, roughness, and stickiness, thereby reducing drinkability. In this study, the composite sensory score declined when the pulp concentration exceeded the selected range, suggesting that excessive pulp adversely affected the overall sensory evaluation. Similar effects have been reported in other fruit juice systems, with increases in suspended solids or processing additives improving certain quality attributes but reducing sensory suitability when the formulation becomes unbalanced [35,36]. This finding is particularly important for A. trifoliata, whose pulp contains polysaccharide-rich components that may interact with suspended particles and added stabilizers. Therefore, the pulp concentration should be controlled not only as a flavor or solids variable but also as a structural factor that may influence the rheological and colloidal behavior of the juice.

Sucrose concentration had the lowest relative influence among the three formulation variables, although it remained important for achieving an acceptable flavor profile. This may be because sweetness can compensate for acidity only within a limited range, whereas the acid level more strongly defines the overall sensory identity of the beverage. Within the orthogonal design, 8% sucrose was associated with the highest-level mean for the composite sensory score. The lower relative influence of sucrose indicates that sweetness adjustment was less influential than acidity within the concentration range evaluated in the present study.

Physical stability is a central quality attribute of cloudy juice, and the present results show that particle-size control was essential in improving the suspension stability of *A. trifoliata* cloudy juice. Finer filtration improved stability, with the highest stability observed under 400-mesh filtration. This can be explained by the reduction in particle size, which decreases sedimentation velocity and promotes a more uniform dispersion of suspended particles. The 100–400 mesh range used in this study is consistent with the U.S. Standard Sieve Series and with particle-size control approaches previously applied in cloudy juice systems [30,31]. However, very fine filtration may also reduce yield, remove desirable pulp components, increase processing time, and raise production costs. Thus, although 400-mesh filtration provided short-term suspension stability under the tested conditions, future process development should evaluate the balance between stability, yield, sensory richness, and processing feasibility [17,37].

The stabilizer screening results further demonstrate that hydrocolloid type strongly affects cloudy juice stability. Pectin showed the strongest individual stabilizing effect, suggesting that it interacted favorably with the *A. trifoliata* juice matrix. Pectin can improve cloud stability by increasing continuous-phase viscosity, forming weak network structures, and contributing to electrostatic or steric stabilization depending on pH and matrix composition. This behavior is consistent with the recognized role of pectin in stabilizing fruit-based systems, particularly apple and citrus products [37,38]. However, the use of pectin alone at higher concentrations was associated with an excessively thick sensory perception and reduced trained-panel sensory performance. This highlights a common challenge in cloudy beverage development: the stabilizer concentration required to maximize physical stability may not be the same as that required for optimal sensory quality. Therefore, sensory evaluation must be integrated into stabilizer optimization rather than treating stability as the only endpoint.

Guar gum and xanthan gum contributed to stabilization through complementary mechanisms. Guar gum is a galactomannan that can enhance viscosity and provide steric stabilization, while xanthan gum is highly effective at increasing viscosity at low concentrations and restricting particle movement. Xanthan gum can also contribute to suspension stability by forming a weak gel-like network in the continuous phase, which helps maintain particles in suspension. However, excessive xanthan gum may produce a slimy or gummy mouthfeel, reducing sensory acceptability. Hydrocolloid systems containing xanthan and guar gum commonly exhibit non-Newtonian shear-thinning behavior, which can provide relatively high apparent viscosity under low-shear conditions while allowing for easier flow under higher shear. Such behavior could be advantageous for the maintenance of suspended particles while retaining processability. However, rheological flow curves were not measured in the present study; therefore, pseudoplastic or shear-thinning behavior of the optimized *A. trifoliata* formulation cannot be confirmed experimentally. The results of the single-factor trials therefore support the need for a compound stabilizer system where each hydrocolloid contributes a specific stabilizing function at a relatively low concentration.

The orthogonal screening identified 0.03% pectin, 0.05% guar gum, and 0.06% xanthan gum as the highest-performing compound–stabilizer combination among those tested. This combination provided the most favorable balance between the suspension stability coefficient and trained-panel sensory score. Its performance may reflect complementary interactions among the three hydrocolloids rather than the action of a single dominant stabilizer. Pectin may provide network-based and electrostatic stabilization, guar gum may enhance steric protection and mouthfeel, and xanthan gum may increase viscosity and reduce particle mobility. Interactions between xanthan gum and galactomannans such as guar gum can also enhance continuous-phase structuring, whereas pectin may further support particle dispersion in the acidic fruit matrix. These mechanisms provide a plausible explanation for the performance of the three-component system, although molecular interactions were not directly measured in this study. Composite stabilizer systems have also been shown to improve turbidity stability and sedimentation resistance in other fruit juice matrices, including guava, pineapple, and orange juices [16,37,39,40]. Recent studies further suggest that hydrocolloid combinations and their integration with processing technologies such as ultrasound or high-shear homogenization can enhance cloud stability and storage performance in fruit beverages [41]. Notably, the concentration of each hydrocolloid in the selected compound formulation was lower than its corresponding concentration selected during the single-stabilizer screening. This may reduce the risk of flavor masking, gum-like aftertaste, and undesirable thickness [42].

The relatively low level of added pectin required in the selected compound formulation may also reflect the intrinsic composition of *A. trifoliata* pulp. The pulp contains natural polysaccharides and pectic substances that may already contribute to the structural network of the cloudy juice. Added pectin, guar gum, and xanthan gum may therefore reinforce an existing matrix rather than create stability entirely from an unstable system. This matrix-specific behavior is important because stabilizer performance cannot be generalized across fruit juices. Differences in pH, soluble solids, ionic strength, particle size, native pectin, protein, phenolic compounds, and pulp composition can all alter hydrocolloid interactions. Thus, the stabilizer system selected in this study should be interpreted as specific to the tested *A. trifoliata* juice matrix and short-term experimental conditions. From an application perspective, this low-dose stabilizer strategy provides a basis for further evaluation of cloudy-juice stability while limiting undesirable changes in mouthfeel and fruit flavor [43].

A further contribution of this study is the integration of fuzzy mathematical sensory evaluation into formulation and stability optimization. Sensory evaluation of novel fruit beverages is often challenging because panelists may lack established reference standards for unfamiliar flavors. This issue is especially relevant for *A. trifoliata*, which has a distinctive aroma and texture that may not be easily compared with common fruit juices. Conventional sensory scoring methods can capture general preference, but they may be influenced by subjectivity, inconsistent interpretation of descriptors, and individual differences among evaluators. Fuzzy mathematical evaluation addresses part of this limitation by converting qualitative judgments into membership values and integrating multiple attributes through weighted evaluation matrices [22,23,24,26,28].

In this study, fuzzy evaluation allowed color, aroma, taste, appearance, mouthfeel, and gum-related flavor attributes to be combined into composite scores. This approach was useful because the selected formulation was not determined from any single sensory attribute. For example, a sample could show good aroma but poor appearance or good stability but excessive viscosity. The fuzzy evaluation framework provided a more systematic basis for comparing such trade-offs. It was particularly valuable in stabilizer optimization, where physical stability and sensory acceptability can move in opposite directions. By integrating both types of indicators, the method supported selection of the combination providing the most favorable balance between short-term suspension stability and trained-panel sensory performance.

Nevertheless, fuzzy mathematical evaluation has limitations. The weighting coefficients assigned to sensory attributes still depend on predefined methodological judgments, and different weighting schemes could alter the final ranking of formulations. Although the trained panel improved consistency, trained-panel results do not necessarily reflect consumer preference in a broader market. In addition, sensory evaluation was conducted under controlled laboratory conditions, whereas real consumer perception may be influenced by serving temperature, packaging, consumption context, sweetness expectations, and familiarity with *A. trifoliata*. Previous studies have also noted that excessive numbers of evaluation indicators or inappropriate weighting can reduce the discriminative capacity of fuzzy sensory models [28,44]. Therefore, future studies should validate the selected formulation using larger affective consumer tests with demographically diverse cohorts and, where appropriate, preference mapping. Such validation would help determine whether the sensory profile identified by trained evaluators corresponds to actual consumer acceptance.

The study also has several limitations related to storage duration, rheological characterization, and bioactive properties. The suspension-stability assessment was limited to three days at room temperature and should therefore be interpreted as a short-term formulation screening rather than evidence of shelf life or commercial stability. For commercial development, it will be necessary to determine whether the optimized cloudy juice remains stable during extended storage under different temperatures, light exposure, and packaging conditions. Future work should monitor sedimentation, turbidity, color changes, browning, viscosity, microbial safety, and sensory quality over time. In addition, although *A. trifoliata* is known to contain bioactive constituents, this study focused mainly on formulation and short-term suspension stability and did not determine total phenolic content, antioxidant activity, or retention of bioactive compounds following formulation and thermal processing. Further analysis of phenolic content, flavonoids, antioxidant activity, the organic acid profile, sugar composition, and nutrient retention during processing and storage is required before functional properties can be attributed to the developed beverage. Previous studies have shown that stabilizers and processing conditions can affect nutrient retention during juice storage, supporting the need for further shelf-life and quality-preservation studies [45].

From an application perspective, the findings provide a practical framework for formulation screening of cloudy juice from *A. trifoliata* and potentially other underutilized fruits with complex pulp matrices. The optimized approach combines pulp adjustment, sugar–acid balancing, particle-size control, and low-dose compound hydrocolloid stabilization. This strategy is valuable because it addresses the major barriers to cloudy juice development: flavor imbalance, sedimentation, phase separation, and unacceptable mouthfeel. Moreover, future optimization could incorporate multi-criteria decision-making models to integrate sensory quality, long-term stability, nutritional value, processing cost, and scale-up feasibility into a unified product-development framework [35]. Such approaches would further support rational formulation design for large-scale production of *A. trifoliata* beverages. Such studies, together with microbial-safety and consumer-validation assessments, will be required before commercial-scale application of the selected formulation can be established.

## 5. Conclusions

This study systematically evaluated the formulation and short-term suspension stability of *Akebia trifoliata* cloudy juice, demonstrating that product quality depends on the coordinated balance between sensory performance and physical stability. Among the formulation variables, citric acid concentration had the greatest influence on the composite sensory score, and 14% pulp, 8% sucrose, and 0.05% citric acid represented the highest-performing combination among the tested formulations. Stability screening further showed that pectin provided the highest individual suspension stability coefficient, whereas compound-stabilizer evaluation identified 0.03% pectin, 0.05% guar gum, and 0.06% xanthan gum as the highest-performing tested combination based on the integrated suspension-stability and sensory score. The use of fuzzy mathematical sensory evaluation provided a structured framework for integrating multiple sensory and stability-related indicators into formulation decisions. Overall, these findings provide a basis for formulation and short-term stability screening of *A. trifoliata* cloudy juice. The present results do not establish long-term shelf life, rheological performance, microbial safety, bioactive functionality, or population-level consumer acceptance; these attributes require further evaluation before commercial application.

## Figures and Tables

**Table 1:** Descriptive criteria used for trained-panel sensory evaluation of the formulation and short-term stability of Akebia trifoliata cloudy juice.

Factor	Levels			
	Excellent	Good	Medium	Poor
Color	Creamy white with uniform and visually appealing juice color	Creamy white; uniform color with minor loss of visual appeal	Pale but uniform with limited visual appeal	Color too pale; uneven, partly darkened
Aroma	Distinct characteristic *A. trifoliata* aroma with no off odor	*Characteristic A. trifoliata* aroma present but weak	Little or no characteristic aroma	Noticeable off odor
Taste	Characteristic flavor with balanced sweetness and acidity and no off flavor	Characteristic flavor with an acceptable sweetness–acidity balance	Weak characteristic flavor or unbalanced sweetness and acidity	Noticeable off flavor and markedly unbalanced sweetness and acidity
Appearance	Homogeneous, with no visible sedimentation or phase separation	Slight sedimentation without distinct phase separation	Visible sedimentation and partial phase separation	Pronounced sedimentation and phase separation
***Sensory evaluation standard for stability of cloudy juices***				
Appearance	Homogeneous, with no visible sedimentation or phase separation	Slight sedimentation without distinct phase separation	Visible sedimentation and partial phase separation	Pronounced sedimentation and phase separation
Mouthfeel	Smooth and readily drinkable	Slightly thick but acceptable	Noticeably thick or sticky	Markedly gummy or excessively sticky
Gum-related flavor	No detectable gum-related off flavor	Slight gum-related flavor	Clearly detectable gum-related flavor	Pronounced gum-related off flavor

**Table 2:** Orthogonal test results of cloudy juice formulations.

Experiment	Factors				Sensory Score
	Original Pulp Content/% A	Sugar Content/% B	Acid Content/% C	Error Column D	
1	1 (12)	1 (6)	1 (0.05)	1	80.6
2	1	2 (7)	2 (0.10)	2	71.6
3	1	3 (8)	3 (0.15)	3	80.4
4	2 (14)	1	2	3	75.2
5	2	2	3	1	82.4
6	2	3	1	2	87.2
7	3 (16)	1	3	2	73.2
8	3	2	1	3	83.8
9	3	3	2	1	71.0
k_1_	77.533	76.333	83.867	78.000	
k_2_	81.600	79.267	72.600	77.333	
k_3_	76.000	79.533	78.667	79.800	
R	5.600	3.200	11.267	2.467	

Note: Sensory scores represent mean composite scores obtained from three independently prepared juice batches (*n* = 3). k_1_, k_2_, and k_3_ represent the mean sensory scores at factor levels 1, 2, and 3, respectively. R denotes the range of mean values among the three levels for each factor. Factors: A—original pulp content (%); B—sugar content (%); C—acid content (%). The error column represents the residual term.

**Table 3:** Orthogonal test results of the cloudy juice stabilizer compounds.

Experiment	Factors				The Stability Coefficient/%	Stability Sensory Score /Points	Composite Score
	Pectin/% A	Guar Gum/% B	Xanthan Gum/% C	Error Column D			
1	1 (0.02)	1 (0.050)	1 (0.04)	1	0.434	85.9	0.435
2	1	2 (0.075)	2 (0.06)	2	0.458	74.9	0.257
3	1	3 (0.100)	3 (0.08)	3	0.679	83.2	0.610
4	2 (0.03)	1	2	3	0.925	89.5	0.951
5	2	2	3	1	0.978	77.8	0.787
6	2	3	1	2	0.483	65.2	0.104
7	3 (0.04)	1	3	2	0.537	71.7	0.271
8	3	2	1	3	0.704	62.0	0.248
9	3	3	2	1	0.958	62.8	0.497
k_1_	0.434	0.552	0.262	0.573			
k_2_	0.614	0.431	0.568	0.211			
k_3_	0.339	0.404	0.556	0.603			
R	0.275	0.148	0.306	0.392			

Note: The suspension stability coefficient is expressed as a ratio from 0 to 1. Each formulation was independently prepared in triplicate (*n* = 3). k_1_, k_2_, and k_3_ represent the mean composite stability scores at factor levels 1, 2, and 3, respectively. Factors: A—pectin (%); B—guar gum (%); C—xanthan gum (%). The error column represents the residual term.

## Data Availability

The original contributions presented in this study are included in the article/Supplementary Materials. Further inquiries can be directed to the corresponding authors.

## Supplementary Materials

The following supporting information can be downloaded at: https://www.mdpi.com/article/10.3390/foods15162911/s1, Supplementary Table S1: Orthogonal test factors and levels for the formulation of cloudy juice of *Akebia trifoliata*. Supplementary Table S2: Variance analysis results of cloudy juice formulations. Supplementary Table S3. Orthogonal test factors and levels of compound stabilizer for cloudy juice of *Akebia trifoliata*. Supplementary Table S4. Variance analysis results of the cloudy juice stabilizer compounds in *Akebia trifoliata*. Supplementary Figure S1. Comparison of juice appearance of Akebia trifoliata under different formulations. Supplementary Table S5. Variance analysis results of cloudy juice stabilizer compounding.
