# Supplementary material for: Optimization of Sensory Quality and Short-Term Colloidal Stability of Akebia trifoliata Cloudy Juice for Beverage Development
Source: Foods. 2026 Aug 20;15(16):2911. doi: 10.3390/foods15162911 (PMC13512717; doi:10.3390/foods15162911)
Supplement: Supplementary file 1 [file foods-15-02911-s001.zip › Supplementary Table and Figures.pdf]

Supplementary Table S1: Orthogonal test factors and levels for the formulation of cloudy juice of *Akebia trifoliata*

Levels	Factors		
	**Original pulp content/%**	**Sugar content/%**	**acid content/%**
1	12	6	0.05
2	14	7	0.10
3	16	8	0.15

Supplementary Table S2: Variance analysis results of cloudy juice formulations

Factors	Deviation sum of squares	Degrees of freedom	F ratio	F critical value	Significance
Original pulp content	50.249	2	5.144	19.000	
Sugar content	18.916	2	1.936	19.000	
Acid content	190.782	2	19.529	19.000	*
Error	9.77	2			

Note: *The asterisk* (*) indicates significance at $p < 0.05$.

Supplementary Table S3. Orthogonal test factors and levels of compound stabilizer for cloudy juice of *Akebia trifoliata*

Levels	Factors		
	**Pectin/%**	**Guar gum/%**	**Xanthan gum/%**
1	0.020	0.050	0.040
2	0.030	0.075	0.060
3	0.040	0.100	0.080

Supplementary Table S4. Variance analysis results of the cloudy juice stabilizer compounds in *Akebia trifoliata*

Factors	Deviation sum of squares	Degrees of freedom	F ratio	F critical value	Significance
Pectin	0.117	2	0.409	19.000	
Guar gum	0.038	2	0.133	19.000	
Xanthan gum	0.180	2	0.629	19.000	
Error	0.29	2			

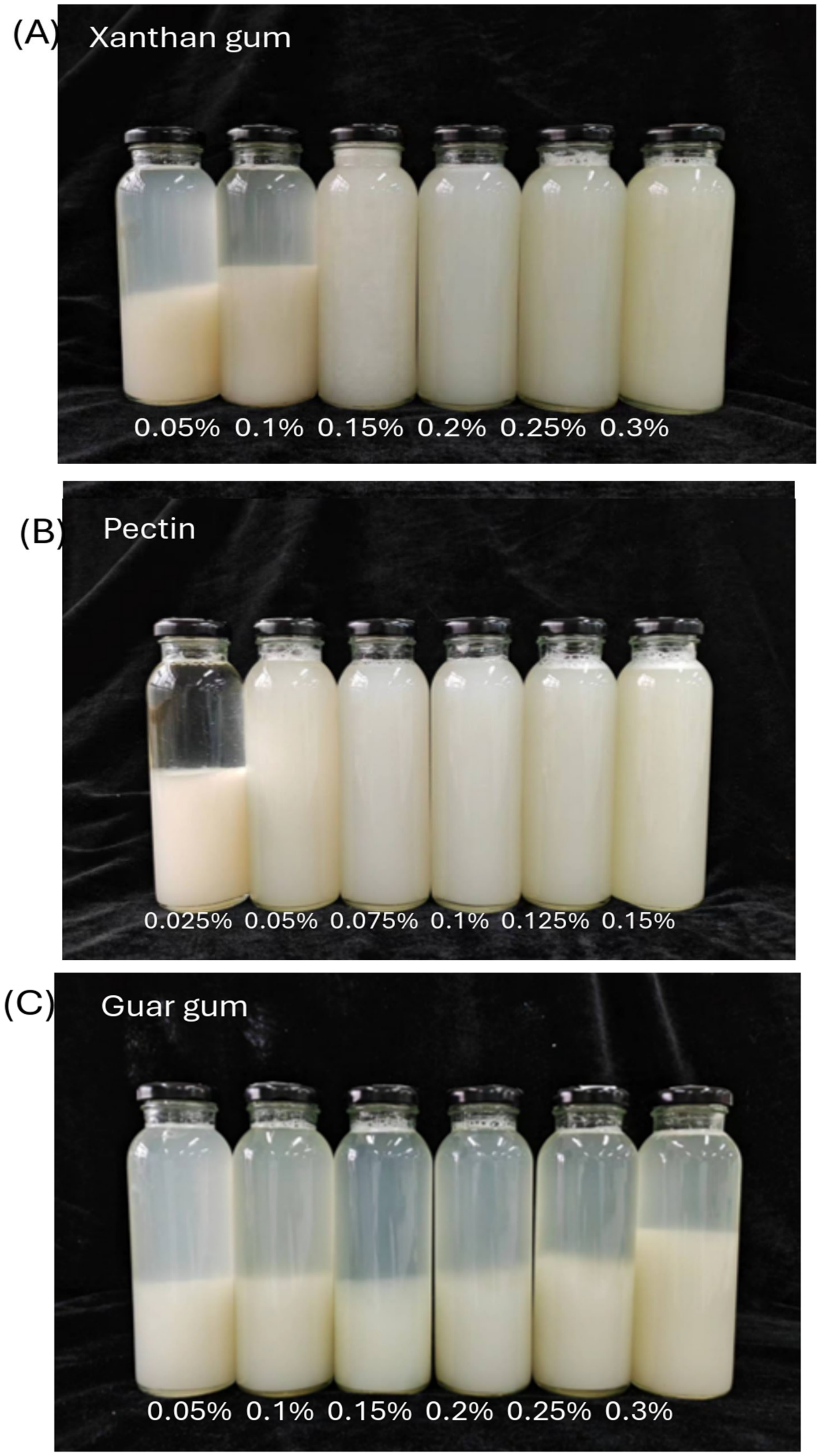

Supplementary Figure S1. Comparison of juice appearance of *Akebia trifoliata* under different formulations.
