# Supplementary material for: Optimization of Sensory Quality and Short-Term Colloidal Stability of Akebia trifoliata Cloudy Juice for Beverage Development
Source: Foods. 2026 Aug 20;15(16):2911. doi: 10.3390/foods15162911 (PMC13512717; doi:10.3390/foods15162911)
Supplement: Supplementary file 1 [file foods-15-02911-s001.zip › Supplementary Table and Questionaire.pdf]

Supplementary Table S5. Variance analysis results of cloudy juice stabilizer compounding

Factors	Deviation sum of squares	Degrees of freedom	F ratio	F critical value	Significance
Pectin	0.117	2	0.409	19.000	
Guar gum	0.038	2	0.133	19.000	
Xanthan gum	0.180	2	0.629	19.000	
Error	0.29	2			

The formulation of *Akebia trifoliata* cloudy juice

One-way test of formulation

**Factors**	**Levels**			
	**Excellent**	**Good**	**Medium**	**Poor**
Color	Creamy white of *A. trifoliata*; uniform color; attractive juice color	Creamy white; uniform color; average level of color attractiveness	Color is too light; color is uniform; color is generally attractive	Color too pale; uneven, partly dark; lack of appetite
Odor	More distinctive aroma of *A. trifoliata*	*A. trifoliata* has a weak aroma	Odorless	Bad odor
Taste	Has a *A. trifoliata* flavor with the right amount of sweetness and acidity	Has a *A. trifoliata* flavor, with a medium sweetness and acidity	No *A. trifoliata* flavor, sweetness and acidity are out of place	Off-flavors are noticeable, and sweetness and acidity are out of place
Appearance	Homogeneous, no delamination	a little sediment	more sediment	Layered and with significant sediments

**Factors**		Color	Odor	Taste	Appearance
original pulp	A1				
	A2				
	A3				
	A4				
	A5				
sugar	B1				
	B2				
	B3				
	B4				
	B5				
citric acid	C1				
	C2				
	C3				
	C4				
	C5				

The formulation of *Akebia trifoliata* cloudy juice

Orthogonal test of formulation

Factors	Levels			
	**Excellent**	**Good**	**Medium**	**Poor**
Color	Creamy white of *A. trifoliata*; uniform color; attractive juice color	Creamy white; uniform color; average level of color attractiveness	Color is too light; color is uniform; color is generally attractive	Color too pale; uneven, partly dark; lack of appetite
Odor	More distinctive aroma of *A. trifoliata*	*A. trifoliata* has a weak aroma	Odorless	Bad odor
Taste	Has a *A. trifoliata* flavor with the right amount of sweetness and acidity	Has a *A. trifoliata* flavor, with a medium sweetness and acidity	No *A. trifoliata* flavor, sweetness and acidity are out of place	Off-flavors are noticeable, and sweetness and acidity are out of place
Appearance	Homogeneous, no delamination	a little sediment	more sediment	Layered and with significant sediments

**Factors**	Color	Odor	Taste	Appearance
1				
2				
3				
4				
5				
6				
7				
8				
9				

The stability of *Akebia trifoliata* cloudy juice

Orthogonal test of stabilizers

**Factors**	**Levels**			
	**Excellent**	**Good**	**Medium**	**Poor**
Appearance	Homogeneous, no delamination	A little sediment	More sediment	Layered and with significant sediments
Taste	Smooth and refreshing	Slightly sticky mouthfeel	Sticky mouthfeel	Stickiness is noticeable
Flavor	Normality	Slight gum flavor	Gum flavor	Pronounced gum flavor

**Factors**	Color	Odor	Taste	Appearance
1				
2				
3				
4				
5				
6				
7				
8				
9				
